# Auguring Fake Face Images Using Dual Input Convolution Neural Network

**DOI:** 10.3390/jimaging9010003

**Published:** 2022-12-21

**Authors:** Mohan Bhandari, Arjun Neupane, Saurav Mallik, Loveleen Gaur, Hong Qin

**Affiliations:** 1 Department of Science and Technology, Samriddhi College, Lokanthali, Bhaktapur 44800, Nepal; 2 School of Engineering and Technology, Central Queensland University, Norman Gardens, Rockhampton, QLD 4701, Australia; 3 Department of Environmental Health, School of Public Health, Harvard University, Boston, MA 02115, USA; 4 Research Assistant, University of Arizona, Tucson, AZ 85721, USA; 5 Amity International Business School, Amity University, Noida 201303, India; 6 School of Computer Science, Taylor University, Subang Jaya 47500, Malaysia; 7 Graduate School of Business, University of South Pacific, Suva 1168, Fiji; 8 Department of Computer Science and Engineering, University of Tennessee, Chattanooga, TN 37996, USA

**Keywords:** Convolutional Neural Network (CNN), deepfakes, face detection, SHAP, XAI

## Abstract

Deepfake technology uses auto-encoders and generative adversarial networks to replace or artificially construct fine-tuned faces, emotions, and sounds. Although there have been significant advancements in the identification of particular fake images, a reliable counterfeit face detector is still lacking, making it difficult to identify fake photos in situations with further compression, blurring, scaling, etc. Deep learning models resolve the research gap to correctly recognize phony images, whose objectionable content might encourage fraudulent activity and cause major problems. To reduce the gap and enlarge the fields of view of the network, we propose a dual input convolutional neural network (DICNN) model with ten-fold cross validation with an average training accuracy of 99.36 ± 0.62, a test accuracy of 99.08 ± 0.64, and a validation accuracy of 99.30 ± 0.94. Additionally, we used ’SHapley Additive exPlanations (SHAP) ’ as explainable AI (XAI) Shapely values to explain the results and interoperability visually by imposing the model into SHAP. The proposed model holds significant importance for being accepted by forensics and security experts because of its distinctive features and considerably higher accuracy than state-of-the-art methods.

## 1. Introduction

Numerous wisecrackers have used deepfake (DF) techniques to create various doctored images and videos featuring well-known celebrities (including Donald Trump, Barack Obama, and Vladimir Putin) making claims they would never make in real-life situations [1]. To more accurately assess the exhibition differences between various locations tactics, several studies examine the presentation contrasts between the few DFs discovery procedures for two-stream, HeadPose, MesoNet, visual artifacts, and multi-task [2].

The incredible advancements that have been made in deep learning (DL) research have made it possible to resolve complex tasks in computer vision [3], including neural network optimization, natural language processing [4], image processing [5], intelligent transportation [6], and image steganography [7]. Machine learning (ML) algorithms have been heavily incorporated into photo-editing software recently to assist with creating, editing, and synthesizing photographs and enhancing image quality. As a result, even those without extensive editing experience in photography can produce sophisticated, high-quality images [8]. Additionally, many photo-editing programs and applications provide a variety of amusing features such as face swapping to draw users. For instance, face-swapping apps automatically identify faces in images and replace one person’s face with an animal or another human.

Face images, such as identifying people, are often used for biometric authentication since they convey rich and simple personal identity information. For instance, facial recognition is used more often in our daily lives for things such as financial transactions, and access management [9]. Face modification technology is advancing quickly, making it easier than ever to create false faces, which hastens the distribution of phony facial photos on social media [10,11]. The inability of humans to discern between real and false faces due to sophisticated technology has led to ongoing worries about the integrity of digital information [12,13]. Different DL models such as the convolution neural network (CNN) are frequently used to build false face detectors to lessen the adverse effects that manipulation technology has on society [14].

Different monitoring approaches are used to identify and stop these destructive effects. However, most earlier research relies on deciphering meta-data or other easily masked aspects of image compression information. Splicing or copy-move detection methods are also useless when attackers use generative adversarial networks (GAN) to create complex fake images. However, little research is available to identify images produced by GANs [15]. High-quality facial image production has been transformed by NVIDIA’s open-sourced StyleGAN TensorFlow implementation. The democratization of AI/ML algorithms has, however, made it possible for malicious threat actors to create online personas or sock-puppet accounts on social media platforms. These synthetic faces are incredibly convincing as real images [16]. In order to extract knowledge from current models, StyleGAN offers a data-driven simulation that is relevant for manufacturing process optimization [17]. On top of that, the proposed study addresses the issue of identifying fraudulent images produced by StyleGAN [18,19].

The main objective of the proposed study is to anticipate and understand fraudulent images, and the major contributions are outlined in the points that follow:

1. A dual branch CNN architecture is proposed to enlarge the view of the network with more prominent performance in auguring the fake faces.
2. The study explores the blackbox approach of the DICNN model using SHAP to construct explanation-driven findings by utilizing shapely values.

## 2. Related Works

### 2.1. Deep Learning-Based Methods

The authors in [20] suggested that to build a generalizable detector, one should use representation space contrasts since DeepFakes can match the original image/video in terms of appearance to a more significant extent. The authors combined the scores from the proposed SupCon model with the Xception network to use the variability from different architectures when examining the features learned from the proposed technique for explainability. Using the suggested SupCon, the study’s maximum accuracy was 78.74%. In a real open-set assessment scenario where the test class is unknown at the training phase, the proposed fusion model achieved an accuracy of 83.99%. According to the authors in [21], a Gaussian low-pass filter is used to pre-process the images; as a result, the ascendancy of image contents can facilitate the detection capability. In a study proposed by Salman et al. [22], the highest accuracy of 97.97% based on dual-channel CNN was detected from GAN-generated images. Zhang et al. [23] utilized the LFW face database [24] to extract a set of compact features using the bag of words approach and then fed those features into SVM, RF, and MLP classifiers to distinguish swapped-face photos from real ones, acheiving accuracies of 82.90%, 83.15%, and 93.55% respectively. Similarly, Guo et al. [25] suggested a CNN model called SCnet to identify deepfake photos produced by the Glow-based face forgeries tool [26]. The Glow model intentionally altered the facial expression in the phony photographs, which were hyper-realistic and had flawless aesthetic attributes where SCnet maintained 92% accuracy. A technique for detecting Deepfakes was given by Durall et al. [27] and was based on an investigation in the frequency domain. The authors created a new dataset called Faces-HQ by combining high-resolution real face photos from other public datasets such as CELEBA-HQ data set [28] with fakes faces. They achieved decent results in terms of total accuracy using naïve classifiers. On the other hand, by utilizing Lipschitz regularization and deep-image prior methods, the authors in [29] added adversarial perturbations to strengthen deepfakes and trick deepfake detectors. However, detectors only managed to obtain less than 27% accuracy on perturbed deepfakes while achieving over 95% accuracy on unperturbed deepfakes. The authors of [30] used each of the different 15 categories to produce 10,000 false photos for training and 500 fake images for validation. They employed the Adam optimizer [31] with a batch size of 24, a weight decay of 0.0005, and an initial learning rate of 0.0001. The proposed two-stream convolutional neural networks were trained for 24 epochs over all training sets, and styleGAN achieved an accuracy of 88.80%.

### 2.2. Physical-Based Methods

Authors revealed the erratic corneal specular points between two eyes in GAN-simulated faces in [32]. They showed how these artifacts are prevalent in first-rate GAN-synthesized face images and continued by describing an involuntary technique for extracting and comparing corneal specular focus for human eyes, arguing the lack of physical/physiological restrictions in GAN models. The overall accuracy of the study was 94%.

### 2.3. Human Visual Performance

After being selected via Mechanical Turk in a study by authors [33], participants (N = 315) received quick instruction with illustrations of both natural and synthetic faces. After that, each participant watched 128 trials containing a single face and had unlimited time to categorize it appropriately. The participant was unaware that half of the faces were real and half were artificial. They were evenly distributed in gender and race among the 128 trials. The overall accuracy was between 50–60%.

## 3. Materials and Methods

### 3.1. Data Collection and Pre-Processing

**Figure 1:**
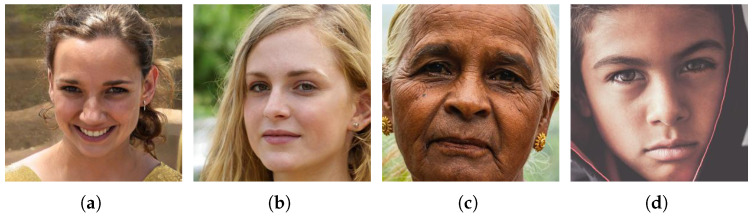
Sample images extracted from the dataset. Note that (**a**,**b**) are fake image, whereas (**c**,**d**) represent real images.

The extraction of a dataset of fake and real face images is from a shareable source [34]. Additionally, the artificial faces created for this dataset using StyleGAN make it more difficult for even a trained human eye to classify them accurately. The real human faces in this dataset gathered to have a fair representation of different features (age, sex, makeup, ethnicity, etc.) encountered in a production setup. Out of 1289 images, 700 are real, whereas the rest are fake. The ratio of train, test, and validation split used was 80:10:10. Some of the samples from the dataset are shown in Figure 1.

Each image was reduced in size to 224 × 224 × 3 to improve the computing performance. Images were shuffled concerning their position to speed up convergence and to prevent the model from overfitting/underfitting, and three epochs of patience (for training accuracy) and early stopping callbacks were imposed. Entire image pixels from the dataset were rescaled into the [0, 1] range.

Even though the dataset had an uneven distribution of classes, the erroneous identification did not result in any greater penalties. Stratified data sampling was used for each training batch to take an equal number of samples from each class.

### 3.2. Proposed Method

**Figure 2:**
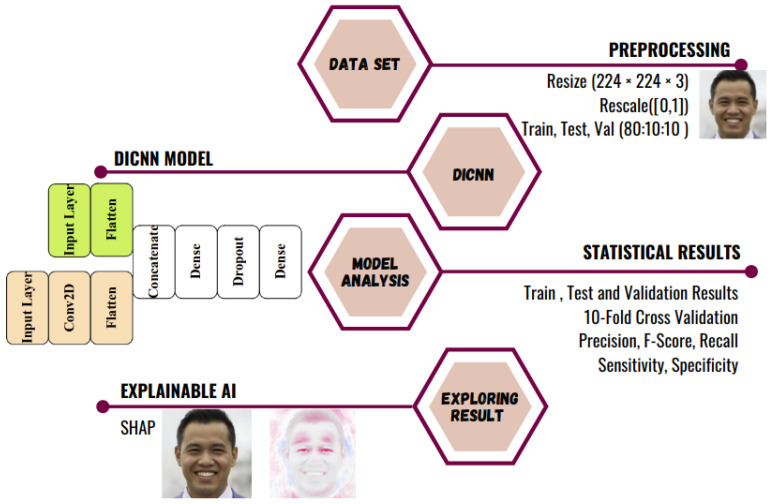
The proposed model to augur doctored images into fake and real.

The bottom-line integrant of the DICNN-XAI approach is: the DICNN model for auguring fake face images and the SHAP-based explanation framework. Figure 2 is the diagrammatic representation of the overall process followed. StyleGAN-generated doctored face images are pre-processed to feed multiple copies into the DICNN model. After the different statistical results of the model are analyzed, it is finally fed into SHAP to explore the blackbox approach of the DICNN model.

#### 3.2.1. Dual Input CNN Model

Inspired by the base model of CNN [35,36], proving the viability of the multi-input CNN model [37,38,39], DICNN-XAI is proposed in the study. To increase robustness, DICNN updates a number of parameters adaptively from numerous inputs [40] and aids in the identification of deep texture patterns [41]. Two input layers (size 224 × 224 ×3) were defined. One branch was continued with a single convolution layer, of which the output was flattened to concatenate the flattened results from the input of another branch. On top of that, two dense layers and dropout layers were added. The overall CNN model architecture is detailed in Table 1.

#### 3.2.2. Explainable AI

Due to the BlackBox nature of DL algorithms as well as due to growing complexities, the need for explainability is increasing rapidly, especially in image processing [42,43,44], criminal investigation [45,46], forensic [47,48,49], etc. Professionals from these sectors may find it easier to comprehend the DL model’s findings and apply them to swiftly and precisely assess whether a face is real or artificial.

SHAP assesses the impact of a model’s features by normalizing the marginal contributions from attributes. The results show how each pixel contributes to a predicted image and supports classification. The Shapley value is computed using all possible combinations of characteristics from dataset images under consideration. Red pixels increase the chance of guessing a class once the Shapley values have been pixelated, while blue pixels make class predictions less likely to be correct [50]. Shapley values are computed using Equation (1).

(1) $$\phi_{i}=\sum_{S\subseteq N\backslash \{i\}}\frac{|S|!(M\;-\;|S|\;-\;1)!}{M!}\left[f_{x}\left(S\cup i\right)\;-\;f_{x}\left(S\right)\right]]$$

For a particular attribute *i*, *f_x_* is the switch of results subsumed by values from SHAP. *S* is the member of all features from feature *N*, with the deviation of feature *i*. The weighting factor $\frac{|S|!(M-|S|-1)!}{M!}$ sums up the numerous ways, and the subset *S* can be permuted. For the attributes with subset *S*, the results are denoted by *f_x_(S*) and are a result of Equation (2).

(2) $$f_{x}\left(S\right)=E\left[f\left(x\right)|x_{S}\right]$$

With each original trait replaced, *(x_i_)*, SHAP replaces a binary variable ($z_{i}^{\prime }$) that represents whether *x_i_* is absent or present as per Equation (3)

(3) $$g(z^{\prime })=\phi_{0}+\sum_{i=1}^{M}\phi_{i}z_{i}^{\prime }=bias+\sum featureContribution$$

In Equation (3), for model *f(x)*, the confined surrogate model is *g*(*z*′).

### 3.3. Implementation

The proposed model is coded in python [51] using Keras [52] and the TensorFlow framework. With 12 GB of RAM in Google Colab [53] and NVIDIA K80 GPU, 10-fold training and testing experiments were performed.

## 4. Results and Discussion

### 4.1. Model Explanation with DICNN

**Figure 3:**
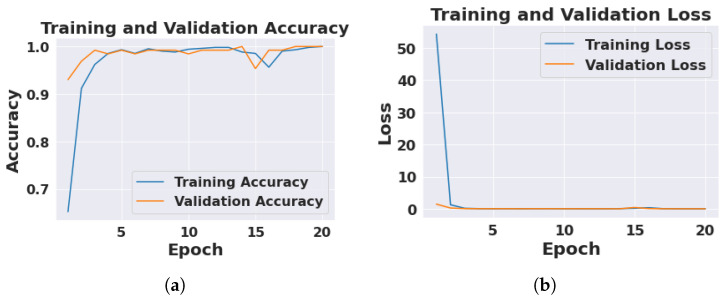
Train and validation results of 10th fold from proposed DICNN. (**a**) Depicts 99.36 ± 0.62% training accuracy and 99.30 ± 0.94% validation accuracy. (**b**) Conveys 0.19 ± 0.31 of training loss and 0.092 ± 0.13 of validation loss.

To evaluate the model, training accuracy, training loss, test accuracy, test loss, validation accuracy, validation loss, and precision, the F1-score and recall were used as conventional statistical metrics. For model training, we defined early termination conditions and a 20-period epoch. The loss and accuracy of DICNN for K = 10-fold is shown in Figure 3. Our DICNN achieved an averaged training accuracy of 99.36 ± 0.62% and a validation accuracy of 99.30 ± 0.94% over the 10-fold (Table 2).

Overall, the suggested DICNN model attains an average test accuracy of 99.08 ± 0.64% and 0.122 ± 0.18 as test loss for K = 10-fold (Table 2).

### 4.2. Model Explanation Using SHAP

**Figure 4:**
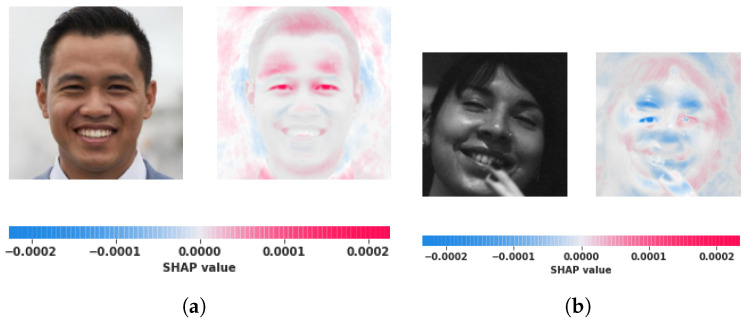
Considering fake and real categories, (**a**) shows the SHAP results for a fake image, whereas (**b**) shows the SHAP results for a real image.

The Shap value that indicates the score for each class is shown as Figure 4. The intensity for red values is concentrated on a fake image, whereas blue values focus on an actual photo. Figure 4a indicates that the image is counterfeit as there are specific manipulations in the eyes and forehead as per the shapely values.

### 4.3. Class-Wise Study of Proposed CNN Model

**Figure 5:**
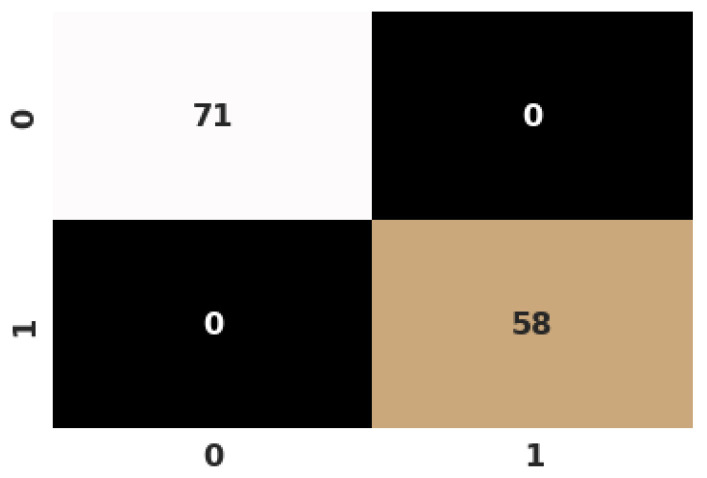
Confusion matrix for test split of 10th fold.

The performance of our suggested model for each class, as well as the accuracy, recall, f1-score, specificity, and sensitivity from K=10-fold data, were studied on a class-by-class basis (Table 3). Looking at the Table 3, it is observed that DICNN achieved a precision of 98.17 ± 2.20–99.23 ± 1.15, a recall of 98.53 ± 0.83–98.77 ± 1.59, an f-score of 98.83 ± 0.98–99.18 ± 0.81, and specificity and sensitivity between 98.41 ± 1.75 and 98.41 ± 1.75. DICNN achieved the highest f-score for the ’Fake’ class, which indicates that the model is susceptible to fake images. In addition, Figure 5 displays the confusion matrix, which shows the accurate and inaccurate classification generated by our model for k = 10-fold.

### 4.4. Comparison with the State-of-the-Art Methods

Table 4 compares the classification performance of our DICNN model with different cutting-edge techniques. We choose the current models based on DL methods, physical-based methods, and human visual performance to make the performance more coherent and pertinent. We select a total of five techniques for comparison. Among the three DL models, our model outperformed two models by 15.37% and 1.39%, whereas another model achieved accuracy by 0.64%. The proposed model’s human visual approach is more accurate by 39.36%, whereas accuracy was higher by 5.36% than the physical approach.

## 5. Conclusions and Future Work

We proposed a DICNN-XAI model with a single convolutional layer for segregating fraudulent face images as real or fake, together with an XAI framework acheiving 99.36 ± 0.62% training accuracy, 99.08 ± 0.64% test accuracy, and 99.30 ± 0.94% validation accuracy over ten-fold. The findings show that DL-XAI models can deliver persuasive artifacts for fake image perception and categorize with high accuracy. The proposed model outperforms other SOTA techniques when classifying fraudulent images alongside XAI.

Only a few images used datasets to train the proposed model, Adam as a optimizer. In the future, the model’s performance may be enhanced by using more complex offline data augmentation techniques, such as the Generative Adversarial Network. XAI can be forced to utilize classification algorithms with higher accuracy and better optimizer. The study could be repeated and used for other XAI algorithms, such GradCAM, to improve auguring problems. Furthermore, algorithms that mimic natural occurrences can be applied to heterogeneous datasets for false imaging modalities, such as the most current developments in computational capacity, deepfake technologies, and digital phenotyping tools [54].

## Figures and Tables

**Table 1:** Summary details for DICNN architecture.

Layer Name	Shape of Output	Param #	Connected to
Input 1	(None, 224, 224, 3)	0	-
Input 2	(None, 224, 224, 3)	0	-
Conv2D	(None, 222, 222, 32)	896	Input 1
Flatten 1	(None, 150,528)	0	Input 2
Flatten 2	(None, 1,577,088)	0	Conv2D
Concatenate Layer	(None, 1,727,616)	0	[Flatten 1, Flatten 2]
Dense 1	(None, 224)	386,986,208	Concatenate Layer
Dropout	(None, 224)	(None, 224)	Dense 1
Dense 2	(None, 2)	450	Dropout
Total params: 386,987,554			
Trainable params: 386,987,554			
Non-trainable params: 0			

**Table 2:** TA, VA, TL, VL, TsA, TsL, and BD for the DICCN model, standing for training accuracy, training loss, validation accuracy, validation loss, test accuracy, test loss, and the number of bad predictions from the model for K = 10-fold in %).

	TA	TL	VA	VL	TsA	TsL	BP
K1	99.90	0.0036	100.00	9.78 × 10 ^−5^	99.00	0.04	0
K2	97.99	0.6236	98.45	0.2445	100.00	0.01	2
K3	99.90	7.84 × 10 ^−4^	100.00	2.11 × 10 ^−5^	99.00	0.09	0
K4	99.61	0.0082	100.00	0.0036	97.67	0.04	0
K5	99.32	0.9420	100.00	1.07 × 10 ^−5^	99.22	0.03	0
K6	98.84	0.1851	97.67	0.3579	99.11	0.62	3
K7	98.74	0.1261	99.22	0.0632	99.22	0.07	1
K8	99.61	0.0122	100.00	0.0014	99.22	0.01	0
K9	99.71	0.0254	97.67	0.2454	98.45	0.30	3
K10	100.00	0.0037	100.00	0.0039	100.00	0.01	0
μ±σ	99.36 ± 0.62	0.19 ± 0.31	99.30 ± 0.94	0.092 ± 0.13	99.08 ± 0.64	0.122 ± 0.18	0.9 ± 1.22

**Table 3:** K = 10-fold results (after 20 epochs, in %): for specificity (Spec), sensitivity (Sen), precision (Pre), F1 score (Fsc), and recall (Rec).

		Spec	Sen	Pre	Fsc	Rec
K1	Fake	99.34	100.00	98.31	99.98	99.15
	Real	100.00	99.34	98.56	98.78	99.56
K2	Fake	97.26	100.00	96.55	98.25	100.00
	Real	100.00	97.26	100.00	98.61	97.26
K3	Fake	100.00	100.00	100.00	99.12	99.34
	Real	100.00	100.00	99.54	98.67	99.76
K4	Fake	99.50	100.00	98.12	99.34	99.89
	Real	100.00	99.50	98.90	99.38	98.86
K5	Fake	100.00	100.00	100.00	100.00	100.00
	Real	100.00	100.00	100.00	100.00	100.00
K6	Fake	96.25	100.00	100.00	98.09	96.25
	Real	100.00	96.25	94.23	97.03	100.00
K7	Fake	96.10	99.25	100.00	99.20	97.34
	Real	99.25	96.10	95.32	98.30	99.89
K8	Fake	100.00	100.00	100.00	100.00	100.00
	Real	100.00	100.00	100.00	100.00	100.00
K9	Fake	95.71	100.00	100.00	97.81	95.71
	Real	100.00	95.71	95.16	97.52	100
K10	Fake	100.00	100.00	100.00	100.00	100.00
	Real	100.00	100.00	100.00	100.00	100.00
μ±σ	Fake	98.41 ± 1.75	99.93 ± 0.23	99.23 ± 1.15	99.18 ± 0.81	98.77 ± 1.59
	Real	99.93 ± 0.23	98.41 ± 1.75	98.17 ± 2.20	98.83 ± 0.98	99.53 ± 0.83

**Table 4:** Comparison of proposed DICNN model with other state-of-the-art methods. ’DL’ and ’Acc’ stand for deep learning and accuracy, respectively.

Ref	Category	Method	Dataset	Performance (%)	XAI
[20]	DL	Xception Network	150,000 images	Acc: 83.99%	No
[21]	DL	CNN	60,000 images	Acc: 97.97%	No
[22]	DL	dual-channel CNN	9000 images	Acc: 100%	No
[23]	DL	CNN	321,378 face images	Acc: 92%	No
[27]	DL	Naive classifiers	Faces-HQ	Acc: 100%	No
[29]	DL	VGG	10,000 real and fake image	Acc: 99.9%	No
[29]	DL	ResNet	10,000 real and fake image	Acc: 94.75%	No
[30]	DL	Two Stream CNN	30,000 images	Acc: 88.80%	No
[32]	Physical	Corneal specular highlight	1000 images	Acc: 94%	No
[33]	Human	Visual	400 images	Acc: 50-60%	No
Ours	DL	DICNN	1289 images	Acc: 99.36 ± 0.62	SHAP

## Data Availability

Publicly available “Fake-Vs-Real-Faces (Hard)”, https://www.kaggle.com/datasets/hamzaboulahia/hardfakevsrealfaces, accessed on 12 October 2022.
